# Atrial Cardiomyopathy and Atrial Fibrillation in Cancer

**DOI:** 10.1155/2021/6685953

**Published:** 2021-02-10

**Authors:** Mengdi Ren, Yu Yao, Xin Yue, Yuye Ning, Yan Yang

**Affiliations:** ^1^Department of Oncology, The First Affiliated Hospital of Xi'an Jiaotong University, No. 277 Yanta West Road, Xi'an 710061, China; ^2^Department of Cardiovascular Medicine, The First Affiliated Hospital of Xi'an Jiaotong University, No. 277 Yanta West Road, Xi'an 710061, China; ^3^Department of Neurology, The First Affiliated Hospital of Xi'an Jiaotong University, No. 277 Yanta West Road, Xi'an 710061, China; ^4^Department of Thoracic Surgery, The First Affiliated Hospital of Xi'an Jiaotong University, No. 277 Yanta West Road, Xi'an 710061, China

## Abstract

The number of patients with oncologic and cardiologic comorbidities is increasing. A growing number of evidence shows an inextricable link between cancer, atrial fibrillation, and atrial cardiomyopathy. Cancer itself and resultant inflammation, anticancer treatment, and other comorbidities lead to atrial remodeling and fibrosis, which increases the tendency to develop atrial cardiomyopathy and atrial fibrillation. The scarcity of current literature and ambiguous results make its relationship difficult to fully understand. In this review, we will summarize existing evidence of the relationships and interactions among cancer, atrial cardiomyopathy, and atrial fibrillation and discuss the underlying mechanisms, and provide better information for the management of these patients.

## 1. Introduction

Cancer patients have better survival nowadays due to multiple emerging therapies such as immunotherapy and target treatment. Therefore, cancer patients are more likely to suffer from cardiovascular disease (CVD) comorbidity. Cancer patients have more than twice the risk of fatal heart disease comparing to the general population [1]. A population-based study of CVD mortality risk shows that cancer patients are at elevated risk of dying from CVDs compared to the general population [2]. Recognition of the interaction between cancer and CVD has shifted from focusing on the cardiovascular toxicity of anticancer therapy [3] to the fact that they may share biological mechanisms that promote both malignancy and CVD development. One of the supporting evidence is that cancer survivors could have more cardiovascular abnormalities than the general population even without exposure to cardiotoxic treatment [4]. This naturally raises a question of whether this relationship is an association or causation between these two diseases, implying a new and exciting research realm in cardio-oncology.

Atrial cardiomyopathy, a term firstly described by Brigden in 1957 [5], affects the atria and atrioventricular system with the potential to produce arrhythmias [6, 7]. The term has evolved for years. EHRA/HRS/APHRS/SOLAECE jointly published a consensus on atrial cardiomyopathies: “any complex of structural, architectural, contractile, or electrophysiological changes affecting the atria with the potential to produce clinically relevant manifestations” [8]. Medical community now agree that atrial fibrillation has strong causality with atrial cardiomyopathy because some instances of genetic diseases provide convincing evidence that underlying atrial tissue abnormalities may be the cause of AF rather than merely impact [9].

Several lines of evidence show that there is an inextricable connection between cancer and atrial fibrillation; however, no study has ever mentioned that this association may also exist in cancer and atrial cardiomyopathy. This review aims to summarize the existing evidence of the relationships and interactions among cancer, atrial cardiomyopathy, and AF, and we discuss the underlying mechanisms and provide useful information to improve the management of these patients.

## 2. Atrial Fibrillation in Cancer

AF is the most common type of heart arrhythmia. Here, we summarize the evidences supporting the relationship between AF and cancer (Table 1). AF can be induced by multiple cancer treatments such as immunotherapy, radiotherapy, surgery, and anticancer drugs [10]. Within 90 days after cancer diagnosis, the risk of AF was highest and this risk decreases over time [11].

Among all treatments, surgery may be the most frequently studied form of cancer-related AF. Several studies suggest that various types of cancer are associated with postoperative AF. A prospective study of 2588 thoracic surgery patients shows that malignant lung or esophagus cancer patients are more likely to develop postoperative AF than patients with benign disease [12]. About 4%–30% of patients after noncardiac surgery for malignancy would develop new-onset AF [12–16]. Meanwhile, the emerging of postoperative AF could predict a poorer long-term survival in lung cancer patients after receiving pulmonary lobectomy [17].But malignant tumor makes patients tending to suffer heavier burden of CVD and more invasive surgery form, making this association requiring more evidence to support.

In addition, cardiotoxicity of AF is a well-recognized adverse effect of certain chemotherapeutic drugs. For example, AF is a common complication induced by anthracyclines with the frequency of 2%–10% [18]. Persistent AF induced by anthracyclines is common and the first episode of AF event often occurs between 8 and 36 months after starting therapy [19]. AF could also occur in patients treated with other anticancer drugs such as fluorouracil, methotrexate, alkylating agents, antimicrotubule agents (docetaxel, paclitaxel), tyrosine kinase inhibitors (imatinib, lapatinib, sunitinib), proteasoma inhibitor (bortezomib), bevacizumab (blocker of the vascular endothelial growth factor), trastuzumab (angiogenesis inhibitor), and immune-checkpoint inhibitors [10, 20–22], and this cardiotoxicity complication has been shown related to poor prognosis. A prospective study investigated 249 lymphoma patients treated with anthracyclines showing that new-onset AF may predict unfavorable outcomes after chemotherapy [19].However, the lack of cardiac monitoring before chemotherapy makes it difficult to distinguish whether there was a preexisting undiagnosed arrhythmia or accompanying arrhythmia caused by chemotherapy. However, some evidenceshows that the incidence of AF is higher in cancer patients even without treatment, which indicates that cancer itself may make patients vulnerable to AF [23].

Meanwhile, recent studies have demonstrated that the manifestation of the arrhythmia could occur preceding the diagnosis of the malignancy, implying that patients of AF are prone to a higher risk of cancer than the general population. In a follow-up cohort study (1980–2011) of 269,742 patients with new-onset AF based on a Danish registry database, 2.5% of the patients were diagnosed with cancer within 3 months, exceeding the expected rate based on national cancer incidence during the period [24]. A similar cohort study including 34,691 initially healthy women also demonstrated that new-onset atrial fibrillation was associated with a higher risk of subsequent cancer diagnosis [25]. A retrospective cohort study of 5130 patients with new-onset AF also confirms this conclusion with a 41% increase in cancer risk compared with the general population [26]. Of note, the risk is highest in the first three months following the diagnosis of AF while the risk declines after that. Thus, the existing evidence cannot support that AF could cause cancer, but only can suggest a correlation. There could be several interpretations for these data. Firstly, occult cancer may exist before patients were diagnosed of AF due to shared corisk factors. Regular medical follow-up and treatment for AF would increase the chance of early detection of potential cancer. Secondly, it is well known that patients of AF are prone to bleeding after anticoagulant drug therapy, which could promote the screening and intervention of early diagnosis of colorectal cancer. It is worth noting that antiarrhythmic drugs such as digoxin have estrogen-like effects and increase the risk of breast cancer in female AF patients [27].

Not all studies are in agreement with this correlation: a population-based, retrospective, matched cohort study suggests that women patients with early breast cancer may not have a higher prevalence of AF before cancer diagnosis [28]. However, such observation should be interpreted with caution since the prevalence of CVD and its risk factors is well known to be lower in women population.

The magnitude and mechanism of the interaction between AF and cancer are still unclear. Proposed mechanisms involved cancer-related inflammation, shared risk factors, anticancer treatment, and other related comorbidities, causing atrial remodeling and increasing the tendency to develop AF for cancer patients [29].

## 3. Atrial Cardiomyopathy in Cancer

There is no direct evidence for the association of atrial cardiomyopathy and cancer; however, several studies may provide some insights in this respect. Recent studies indicate that some embolic strokes of unknown source (ESUS) cases result from subclinical AF and atrial cardiomyopathy [30]. About 50% of cancer-associated strokes are ESUS [31]. A large population-based cohort study suggests that some cryptogenic strokes may be caused by occult cancer [32]. Hence, the stroke events of cancer patients may relate to subclinical AF and atrial cardiomyopathy.

Left atrial enlargement (LAE) on echocardiogram, evidence of left atrial abnormality demonstrated by increased p-wave terminal force in lead V1 (PTFV1) on ECG, and increased serum levels of a form of brain natriuretic peptide (NT-proBNP) and other markers for atrial disease have been used to define atrial cardiomyopathy [30]. The abnormality of these markers has been shown to be related to the cardiotoxicity and prognosis of cancer patients (Table 2).

LA enlargement and dysfunction may relate with higher risk of cardiotoxicity during therapy in breast cancer [33, 34]. Furthermore, a retrospective study including 92 therapy-naive cancer patients and their matched controls suggests that LA reservoir and functions are deteriorated in the cancer group [35]. Peak atrial longitudinal strain decline is a useful indicator of cancer therapeutics-related dysfunction in patients of breast cancer [36].

ECG abnormalities are common among cancer survivors, which can predict cardiac-cause mortality [37]. Although the major abnormalities are isolated ST/T wave abnormalities (7.2%), evidence of myocardial infarction (3.7%), and left ventricular hypertrophy with strain pattern (2.8%) in this study, the markers for assessing atrial mechanical dysfunctions can also be detected in cancer patients. Moreover, left intraatrial and interatrial electromechanical intervals were prolonged in patients with breast cancer after anthracycline therapy [34]. A retrospective case-control study of chronic lymphocytic leukemia patients treated with ibrutinib indicated that left atrial abnormality identified by EKG is a predictor of atrial fibrillation [38]. In addition, the increasing size of left atrial myxoma brings about the broad negative P terminal force in lead V1 (PTFV1) [39].

NT-proBNP is a common and valuable marker regarding not only cancer but also therapy-related cardiac damage or prognosis. Firstly, NT-proBNP could be induced by oncologic diseases (such as invasive squamous cell carcinoma, malignant pericardial effusion, and small cell lung cancer) or related proinflammatory cytokines without cardiac failure [40, 41]. Secondly, NT-proBNP is an independent predictor of malignancies [42, 43]. Its levels are related to disease severity of multiple myeloma (MM) without cardiac disease [44]. A study shows that NT-proBNP levels increased in patients with differentiated thyroid carcinoma and is associated with an elevated risk of cardiovascular events [45].Furthermore, some studies discovered a potential value of NT-proBNP as biomarker for cardiovascular events in cancer during anticancer therapy [43]. Normally NT-proBNP level could increase in cancer patients' plasma within 24 hours after the starting of chemotherapy without significant changes in the echocardiographic parameters and clinical sign [46, 47]. The persistence of increased levels of NT-proBNP after the treatment may be helpful for the detection of patients with high risk of cardiotoxicity [47]. In addition, NT-proBNP was an independent indicator of survival time in patients of non-Hodgkin lymphoma [48] and a predictor for the progression of metastatic renal carcinoma [49].

The pathophysiologic mechanisms underlying the abnormal markers in cancer patients remain unclear. While it is frequently thought to be anticancer therapy-induced, cancer survivors without treatments can also present with abnormalities of these markers. Since there is no report on cancer patients complicated with atrial cardiomyopathy, it could raise a question of whether there is an underlying association between them. Existing research enrolled limited number of patients with various types of cancer and adopted different types of treatment in most studies. Well-standardized studies will be needed to better define the role of atrial cardiomyopathy and related markers in cancer.

## 4. Mechanisms of Increased AF and Atrial Cardiomyopathy in Cancer

As mentioned above, the risk of developing AF is increased for patients with cancer due to shared risk factors, treatments, and disease itself [50].

Oncologic and cardiologic diseases share many risk factors, such as advanced age, obesity, diabetes, and smoking, making the number of patients with comorbidities constantly increasing [51–54].

Antitumor therapy, including surgery, medication, and radiation, can result in atrial fibrillation. The exact mechanisms remain unclear, though it has been proposed that inflammation and apoptosis may be the decisive factors of cardiotoxicity during the treatment [29]. Fibrosis is a consequence of a nonspecific response to cardiomyocyte necrosis or apoptosis [55]. Anticancer therapy may contribute to AF through atrial fibrosis by apoptosis and inflammation.

For therapy-naïve cancer patients with increased incidence of AF, one alternative explanation is that proinflammatory states resulting from cancer itself can promote atrial fibrillation through atrial restructuring [23, 56–58]. Supportive evidence is that circulating levels of CRP, a marker representing the inflammatory state in cancer patients, is not only associated with the presence of AF but can also predict the risk of future development of AF [56, 59]. In addition, pain, malnourishment, infections, and metabolic abnormalities are prevalent in patients with cancer and can result in dysregulated autonomous nervous system, which could also contribute to AF [52, 60]. Moreover, tumors or metastases adjacent to atrial tissues can directly cause AF by compressing the left atrium [61].

Over the past years, the investigation of AF has yielded fundamental insights into the pathophysiology of the electrical, mechanical, and structural abnormalities of the atrium [62]. The fundamental characteristic of the structural pathology associated with AF is atrial fibrosis and structural remodeling [55, 63]. Atrial cardiomyopathy associated with AF includes myocyte degeneration and fibrotic changes of the connective extracellular matrix [55]. Therefore, it can be considered that atrial cardiomyopathy is the substrate for AF. As the atrial cardiomyopathy progresses, atrial dysfunction and eventually the AF develop [63]. Potential factors known to promote atrial fibrosis include aging, inflammation, and oxidative stress, which also could occur in cancer patients [63].

**Figure 1:**
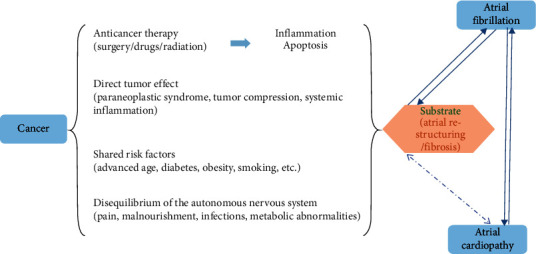
Schematic overview of the link between cancer, atrial fibrillation, and atrial cardiomyopathy. Shared factors may predispose to a comorbidity state of cancer, atrial cardiomyopathy, and atrial fibrillation. Cancer may cause atrial fibrillation and atrial cardiomyopathy by anticancer therapy, autonomous nervous system (ANS) imbalance, direct tumor effect, and other abnormalities. These factors may have direct effects on the left atria (LA) substrate and lead to systemic inflammation and apoptosis. Then, they promote or result in fibrosis and structural remodeling, leading new-onset or existing atrial cardiopathy progress to atrial fibrillation.

Based on the abovementioned theory, we can reasonably infer that atrial cardiomyopathy, AF, and cancer may interplay with each other on pathophysiological levels (Figure 1). Firstly, shared risk factors make cancer patients a high-risk group of atrial cardiomyopathy. Secondly, cancer itself and anticancer therapy may have direct effects on the LA substrate mediated by resultant systemic inflammation and apoptosis. Then, this pathological state would promote or result in fibrosis and structural remodeling of LA, which leads new-onset or existing atrial cardiomyopathy progress to atrial fibrillation.

More studies are needed to explore the interaction between Cancer, AF, and atrial cardiomyopathy, which will provide crucial information on more individualized treatments.

## 5. Challenges and Managements

Given the increasing occurrence of the coexistent CVD in cancer patients, challenges of therapeutic strategies and management are vaster and more complicated than expected. The status of comorbidities and the deleterious effects of anticancer treatments often contribute to less effective treatment, poor life quality, and decreased survival.

The first problem is anticoagulation. Although it is recognized that cancer can lead to hypercoagulable state, the exact effect of cancer on thrombotic risk in patients with AF remains unknown [29]. The clinical recognition of atrial cardiomyopathy suggests a potential value on the identification of individuals at risk of stroke [64] and assessment of novel interventions designed for the prevention of AF [63]. Both atrial cardiomyopathy and cancer are involved in the prethrombotic state. A study showed that cancer patients, whether or not having AF, have an elevated risk of stroke than the general population [65]. However, the existing risk-models that aid to starting anticoagulant therapy do not take the malignant tumor into account. Although the exact extent is not clear now, further related studies are needed to provide some insights in this respect. The clinical recognition of atrial cardiomyopathy in the cancer patients may help with better identification of high-risk patients with hypercoagulable state, which will improve their quality of life and overall survival.

Besides, the presence of CVD comorbidity would affect the clinical decision of cancer treatment and prognosis. Cancer patients with higher risk require cardiologic specialists' review and benefit assessment of anticancer therapy [66]. Multidisciplinary treatment (MDT) including both oncologic and cardiologic specialists would be best choice for patients of such comorbidity.

## 6. Conclusion and Prospect

With the increasing number of cancer patients with CVD, oncocardiology has become an emerging medical subspecialty focusing on cardiovascular effects of cancer and its treatment [67]. Even though the interaction of AF, atrial cardiomyopathy and cancer has been widely documented, the exact mechanism is still unclear. Cancer, possibly through inflammation or effects of the autonomic nervous system, predisposes patients to atrial cardiomyopathy and AF via atrial remodeling and fibrosis. Common risk stratification tools of anticoagulant therapy currently do not take cancer into account as a variable. The clinical value of looking into the atrial cardiomyopathy will provide new insights of this discipline but also the individualized treatment of disease, which will have meaningful implications for future anticancer and supportive treatment.

## Figures and Tables

**Table 1:** Epidemiological evidence of AF in patients with cancer.

Author	Cancer type	Study type	Patient number	Treatment	Correlation or hypothesis
Ji-Hyun Chin	Esophageal cancer	Retrospective observational study	583	Esophagectomy	Postoperatively developed AF was associated with mortality in esophageal cancer patients after esophagectomy
Satoshi Higuchi	Head and neck; lung cancer; gastrointestinal cancer	Prospective cohort study	799	Noncardiac surgery for definitive/suspected malignancy	Perioperative atrial fibrillation in noncardiac surgery was strongly associated with perioperative complications
Ara A. Vaporciyan	Thoracic cancer	Prospective study	2588	Thoracic surgery	The overall incidence of atrial fibrillation was 12.3%
Chung-Wah Siu	Colorectal cancer	Retrospectively study	563	Elective abdominal surgery	4.4% patients developed postoperative AF
Andrea Imperatori	Lung cancer	Retrospectively cohort study	454	Pulmonary lobectomy	AF predicts poorer long-term outcome in 5-year survivors

**Table 2:** Atrial cardiomyopathy associated markers in patients with cancer

Marker	Cancer	Anticancer therapy	Correlation	Reference
Left atrial enlargement	HER2-positive breast cancer	Trastuzumab (TZ) therapy	LA dilatation associated with the development of cardiotoxicity	Corinna Bergamini
	Breast cancer	Anthracycline therapy	Maximum LA volume significantly increased in the patients	Yalin Tolga Yaylal
	Solid cancer (gynecological, breast, gastrointestinal, sarcoma, lungs)	Therapy-naive	LA reservoir and conduit functions were deteriorated in the cancer group	Marijana Tadic
	Breast cancer	Chemotherapy and trastuzumab therapy	Left atrial longitudinal strain as a predictor of cancer therapeutics-related cardiac dysfunction	Hyukjin Park

ECG abnormalities	Breast cancer	Anthracycline therapy	Left intraatrial and interatrial electromechanical intervals were prolonged	Yalin Tolga Yaylal
	Chronic lymphocytic leukemia (CLL)	Ibrutinib	Left atrial abnormality identified by EKG can identify patients at increased risk for this toxicity.	
	Multiple cancer	Cardiac-directed radiation; anthracycline and/or alkylating chemotherapies	ECG abnormalities are common among childhood cancer survivors and predictive of both cardiac and all-cause mortality	Daniel A. Mulrooney
	Left atrial myxoma	Tumor excision	Increased PTFV1 correlates with the tumor size	Norihiro Komiya

NT-proBNP	Coronary artery disease free of cancer	—	NT-proBNP is an independent predictor of malignancies in patients with CAD	José Tuñón
	Neuroendocrine tumor (NET)	—	NT-proBNP are important markers in the diagnosis and survival	Catharina M. Korse
	Multiple myeloma (MM)	Chemotherapy	Elevated levels of NT-proBNP are associated with disease severity	Noemi Pavo
	Differentiated thyroid carcinoma	Total thyroidectomy and radioiodine ablation	NT-proBNP associated with an increased risk for cardiovascular events and all-cause mortality	Esther N. Klein Hesselink
	Cancer	—	BNP levels are elevated and an indicator of heart failure	Sachiko Bando
	Breast cancer	Not-high-dose chemotherapy	NT-proBNP detects high risk of developing cardiotoxicity	S. Romano
	Non-Hodgkin lymphoma	Chemotherapy	NT-proBNP is a marker for risk assessment for NHL patients	Eva Gimeno
	Metastatic renal cell carcinoma	Sunitinib	NT-proBNP predicts for clinical benefit to sunitinib treatment	Konstantinos T. Papazisis
